# The protective effect of lactoferrin on ventral mesencephalon neurons against MPP^+^ is not connected with its iron binding ability

**DOI:** 10.1038/srep10729

**Published:** 2015-06-02

**Authors:** Jun Wang, Mingxia Bi, Huiying Liu, Ning Song, Junxia Xie

**Affiliations:** 1 Department of Physiology, Shandong Provincial Key Laboratory of Pathogenesis and Prevention of Neurological Disorders, Shandong Provincial Collaborative Innovation Center for Neurodegenerative Disorders and State Key Disciplines: Physiology, Medical College of Qingdao University, Qingdao 266071, China; 2 Class 11, Grade 2012, Medical College of Qingdao University, Qingdao 266071, China

## Abstract

Lactoferrin (Lf) can bind to lactoferrin receptor (LfR), leading to iron transport through the plasma membrane. Besides iron transportation, Lf also has antioxidant and anti-inflammatory properties. In the brain, Lf is only synthesized by activated microglia. LfR is present in blood vessels and nigral dopaminergic neurons. Both nigral iron accumulation and microglia activation is believed to be involved in Parkinson’s disease (PD), moreover, increased Lf and LfR in dopaminergic neurons were found in PD cases and MPTP-intoxicated mice. How iron influences microglia to release Lf? Does Lf tend to transport iron to dopaminergic neurons leading to cell death or to protect dopaminergic neuron from neurotoxin? In this study, we observed that iron increased Lf synthesis in activated microglia. In ventral mesencephalon neurons, both iron-free Lf (apo-Lf) and iron-saturated Lf (holo-Lf) exerted neuroprotective effects against MPP^+^ by mechanisms, believed to enhance the mitochondrial transmembrane potential, improve Cu/Zn-superoxide dismutase activity, increase Bcl-2 expression. Although apo-Lf but not holo-Lf chelated cellular iron, there was no difference between the two types of Lf in the neuroprotection. Our data indicate that iron overload increases the activated microglia releasing Lf. Lf plays protective role on ventral mesencephalon neurons against MPP^+^, which is iron-chelating independent.

Parkinson’s disease (PD) is a common neurodegenerative disorder characterized symptomatically by resting tremor, rigidity, and bradykinesia. Neuropathological hallmarks of the disease include the loss of dopaminergic neurons in the substantia nigra (SN) and the subsequent dopamine depletion in the striatum. The exact pathogenesis of PD has not been revealed yet. Extensive studies have demonstrated that iron plays a key role in the pathogenesis of PD. Iron levels increase in the SN of patients with PD and animal models but not in other brain regions^1^. Iron is a potential toxin based on the capacity of excessive iron to react with hydrogen peroxide to produce hydroxyl radicals leading to cell death^2^. PD patients and various cellular and animal models of PD strongly suggest that the activated microglia plays a prominent role in mediating the progressive neurodegenerative process. These activated microglia showed amplified levels of iron deposit, however, the relationship between microglia activation and iron accumulation was not fully elucidated.

Lactoferrin (Lf), an iron-binding protein belonging to the transferrin family, can bind and transfer Fe^3+^ ions, playing a key role in iron metabolism. It is produced by the exocrine glands and is widely distributed in all body fluids such as tears, saliva, milk and neutrophilic leukocytes^3^. Lactoferrin receptor (LfR) has been shown to be present at the surface of different tissues and cell types, participating in Lf internalization by a receptor-mediated pathway. In the brain, Lf is only synthesized and released by activated microglia^4^. LfR is present in blood vessels and nigral dopaminergic neurons^5^, which suggests that Lf and LfR may be involved in iron penetration into brain parenchyma, as well as to the cytoplasm of dopaminergic neurons. Moreover, an increase in Lf staining within nigral neurons was found in PD cases and MPTP-intoxicated mice^4^. Interestingly, in the SN, LfR immunoreactivity was increased on both dopaminergic neurons and microvasculature in patients with PD compared to healthy control. And this increase was highest in the most severely affected dopaminergic cell groups, suggesting a relationship between Lf/LfR increase and dopaminergic neurons degeneration. Thus, the increased Lf and LfR on dopaminergic neurons in PD may account for the excessive accumulation of iron. However, Lf also has a wide variety of biological functions, for example, antioxidant activities, anti-carcinogenic and anti-inflammatory properties, many of which do not appear to be connected with its iron binding ability^6^,^7^. Then there are three questions need us to clarify: 1) Both iron and activated microglia were increased in SN of PD patients, then how iron influences the function of microglia to release Lf? 2) What is the role for the up-regulations of Lf and LfR on the dopaminergic neurons in PD, does the Lf tend to transport iron to dopaminergic neurons leading to cell death or to protect dopaminergic neuron from neurotoxin. 3) There are mainly two forms of Lf: iron-free (apo-Lf) and iron-saturated (holo-Lf). Although apo-Lf is the main form that microglia released, we still want to know if the iron-saturated holo-Lf is harmful to the dopaminergic neurons?

In this study, we investigated how iron levels affected the Lf release in activated microglia, observed the effects of Lf on primary cultured ventral mesencephalon (VM) neurons against 1-methyl-4-phenylpyridinium ion (MPP^+^). The results show that iron overload can increase the activated microglia releasing Lf. Apo-Lf plays protective role on VM neurons against MPP^+^, even when Lf is iron-saturated as holo-Lf, it is still protective on VM neurons. The protective effect of Lf on VM neurons against MPP^+^ does not appear to be connected with its iron binding ability. The present study provides powerful evidence on etiology and promising strategies on prevention and therapy of PD.

## Methods

### Animals and chemical reagents

Spraque-Dawley rats were given free access to food and water, and kept in a 12-h light/dark cycle. All procedures were carried out in accordance with the National Institutes of Health Guide for the Care and Use of Laboratory Animals and were approved by the Animal Ethics Committee of Qingdao University. MPP^+^, ferric ammonium citrate (FAC), Deferioxamine (DFO), Rhodamine123 (Rh123), MPTP and primary antibody against LfR, Cu/Zn-superoxide dismutase (Cu/Zn-SOD), Bcl-2, Bax, were from Sigma Chemical Co. (St Louis, MO, USA). Apo-Lf and holo-Lf were from Antibodies online (Germany). Calcein-AM was from Molecular Probes (Molecular Probes Inc., Carlsbad, CA, USA). Dulbecco’s modified Eagle’s medium (DMEM)/F12 and B27 were from Gibco (Grand Island, NY, USA). All other chemicals and regents were of the highest grade available from local commercial sources.

### Microglia culture

Primary microglia were isolated from newborn Spraque-Dawley rats according to a previous study^8^,^9^, with some modifications. Briefly, cerebral cortices were dissected from 1-day-old Sprague Dawley rats, stripped of the meninges and mechanically dissociated with a pipette until it is dispersed. After centrifugation, cells were suspended in DMEM/F12 supplemented with 10% fetal bovine serum (FBS), 100 units/ml penicillin, and 100 μg/ml streptomycin and seeded in poly-D-lysine-coated 150 cm^2^ flasks (5 brains/flask). Cells were grown in a humidified atmosphere of 5% CO_2_ at 37 °C. After 14 days, microglia were harvested in culture media containing serum by shaking the flasks at 190 rpm for 2 h. After centrifugation, the cell suspension was plated on 12-well plates at a density of 1.2 × 10^6^ cells/ml for further experiments as described below. The purity of the microglial cultures was greater than 95% based on immunofluorescence staining with a specific microglial marker CD11b (see Supplementary Fig. S1 online).

### VM neuron culture

VM neurons were obtained from embryonic Spraque-Dawley rat mesencephalon as described previously^10^,^11^, with some modifications. Briefly, regions of the VM were dissected from embryonic 14-day rat brains with a mild mechanical trituration. After centrifugation, cells were seeded to 12-well (1 × 10^6^/well) culture plates precoated with poly-D-lysine (20 μg/ml) and maintained in DMEM/F12 supplemented with 10% fetal bovine serum (FBS), 100 units/ml penicillin, and 100 μg/ml streptomycin. Cells were grown in a humidified atmosphere of 5% CO_2_ at 37 °C for 18 h, and then the culture medium was changed to serum free DMEM/F12 supplemented with 2% B27. Cells were grown for a further 4 days before use. Neuron purity was about 95% based on immunofluorescence staining with a specific neuron marker microtubule-associated protein 2 (MAP2) (see Supplementary Fig. S2 online).

### Enzyme-linked immunoabsorbent assay (ELISA) of Lf

Microglia were seeded in 12-well plates, pre-incubated for 12 h with 100 μmol/L FAC, and then stimulated for another 24 h with 100 μmol/L MPP^+^. Culture supernatants were collected, and the concentrations of Lf were determined using ELISA kits (Bethyl Laboratories, USA) as described in the manufacturers’ instructions.

### Total RNA extraction and quantitative PCR

Microglia were treated as described above. Cells were lysed and RNA extracted using Trizol Reagent (Invitrogen) according to the manufacturer’s instructions. Then 5 μg total RNA was reverse transcribed in a 20 μL reaction using a reverse-transcription system (Promega). Quantitative PCR was employed to detect the expression of Lf. TaqMan probe and primers were designed using the default settings of Primer Express 2.0 (PE Applied Biosystems). Each set of primers was used with a TaqMan probe labeled at the 5′- end with 6-carboxyfluorescein (FAM) reporter dye and at the 3′- end with 6-carboxy-tetrame-thylrhodamine (TAMRA) quencher dye. The following primers and probes were employed: Lf forward 5′-CCGGATTCACTATTATGC-3′, reverse 5′-CTTCCTCAAGGGATACAG-3′, and probe 5′-CAGCAGTGACATTCGTCTGAACC-3′;GAPDH forward 5′- CCCCCAATGTATCCGTTGTG -3′, reverse 5′- GTAGCCCAGGATGCCCTTTAGT -3′, probe 5′- TCTGACATGCCGCCTGGAGAAACC-3′. Amplification and detection were performed with the following conditions: an initial hold at 95 °C for 10 s, followed by 40 cycles at 95 °C for 5 s and 60 °C for 45 s.

### Flow cytometric measurement of mitochondrial transmembrane potential (ΔΨm)

The changes of ΔΨm with various treatments in VM neurons were measured by rhodamine123 (Rh123) using flow cytometry (Becton Dickinson, USA) as described previously^12^. Rh123 assay is based on the selective accumulation of this dye in the mitochondria by facilitated diffusion. When ΔΨm is decreased, the amount of Rh123 that enters the mitochondria is decreased as there is no facilitated diffusion^13^. Thus, the uptake of Rh123 into mitochondria is an indicator of ΔΨm. VM neurons were seeded in 12-well plates and after attachment they were treated with 100 ng/ml apo-Lf or 100 ng/ml holo-Lf for 4 h followed by100 μmol/L MPP^+^ for 24 h. To block binding of ligands to LfR, VM neurons were treated with Anti-LfR for 1 h at 37 °C. Then, cells were treated as above. After washing with HBS for three times, Rh123 (5 μmol/L) was added and incubation was performed for 30 min at 37 °C. The cells were re-suspended in 1 ml HBS. For analysis, fluorescent intensity was recorded at 488 nm excitation and 525 nm emission wavelengths (Fluorescence 1, FL1). Results were demonstrated as FL1-H (Fluorescence 1-Histogram), setting the gated region M1 and M2 as a marker to observe the changing levels of fluorescence intensity using Cellquest Software.

### Western blots

After pretreatment with 100 ng/ml apo-Lf or 100 ng/ml holo-Lf, VM neurons were incubated with 100 μmol/L MPP^+^ for 24 h. To examine the expressions of LfR, Cu/Zn-SOD, Bcl-2 and Bax, cells were digested with RIPA lysis buffer (50 mM Tris-HCl, 150 mM NaCl, 1% Nonidet-40, 0.5% sodium deoxycholate, 1 mM EDTA, 1 mM PMSF) with protease inhibitors (pepstatin 1 μg/mL, aprotinin 1 μg/mL, leupeptin 1 μg/mL) for 30 min. The lysate was centrifuged at 12,000 g for 20 min at 4 °C, and the supernatant was used for analysis. Protein concentration was established using the BCA protein assay kit (Beyotime, Jiangsu, China). A total of 25 μg of protein from each sample was separated using 10% SDS-polyacrylamide gels and transferred to PVDF membranes. After 2 h blocking with 10% non-fat milk at room temperature, the membranes were incubated with rabbit-anti-rat LfR (1:6000), Cu/Zn-SOD (1:10000), Bcl-2 (1:1000), Bax (1:1000) antibodies over night at 4 °C. Anti-rabbit secondary antibody conjugated to horseradish peroxidase was used at 1:10000 (Santa Cruz Biotechnology, Santa Cruz, CA). Blots were also probed with anti-β-actin antibody (1:10000) as a loading control. Cross-reactivity was visualized using ECL Western blotting detection reagents and analyzed by scanning densitometry using a UVP Image System (Upland, CA, USA).

### Labile iron pool (LIP) measurement

LIP is a pool of chelatable and redox-active iron, which acts as a self-regulatory pool that is sensed by cytosolic iron regulatory proteins (IRPs), and its feedback is regulated by IRP-dependent expression of iron import and storage proteins^14^. LIP can be quantified based on its ability to quench the fluorescence of calcein and the ability of high-affinity specific chelators such as salicylaldehyde isonicotinoyl hydrazone (SIH), deferiprone and DFO to bind and remove it from calcein and thereby increase the fluorescence emitted by the cells^15^. After pretreated with 100 μmol/L FAC and 100 μmol/L MPP^+^ for 24 h, VM neurons were washed twice with HBS and incubated with 0.5 μmol/L calcein-AM for 30 min at 37 °C. Then, the cells were washed twice and treated with 100 ng/ml apo-Lf ,100 ng/ml holo-Lf or 100 μmol/L DFO for the subsequent 30 min, 1 h, 4 h, 12 h and 24 h. Following staining and washing with HBS, cells were analyzed by flow cytometry at different time points as above. Cells were passed at a rate of about 1000/s. Calcein was exited at 488 nm and fluorescence was measured at 525 nm, using the FL1 PMT with logarithmic amplification as previously described^16^.

### Statistical analysis

Results are presented as means ± S.E.M. One-way analysis of variance (ANOVA) followed by the Student-Newman-Keuls test was used to compare difference between means in more than two groups. LIP measurements were carried out using two-way ANOVA followed by the Student-Newman-Keuls test. Data are presented as means ± S.E.M. A probability of P < 0.05 was taken to indicate statistical significance.

## Results

### Lf release and mRNA expression were enhanced in activated microglia with iron load

**Figure 1:**
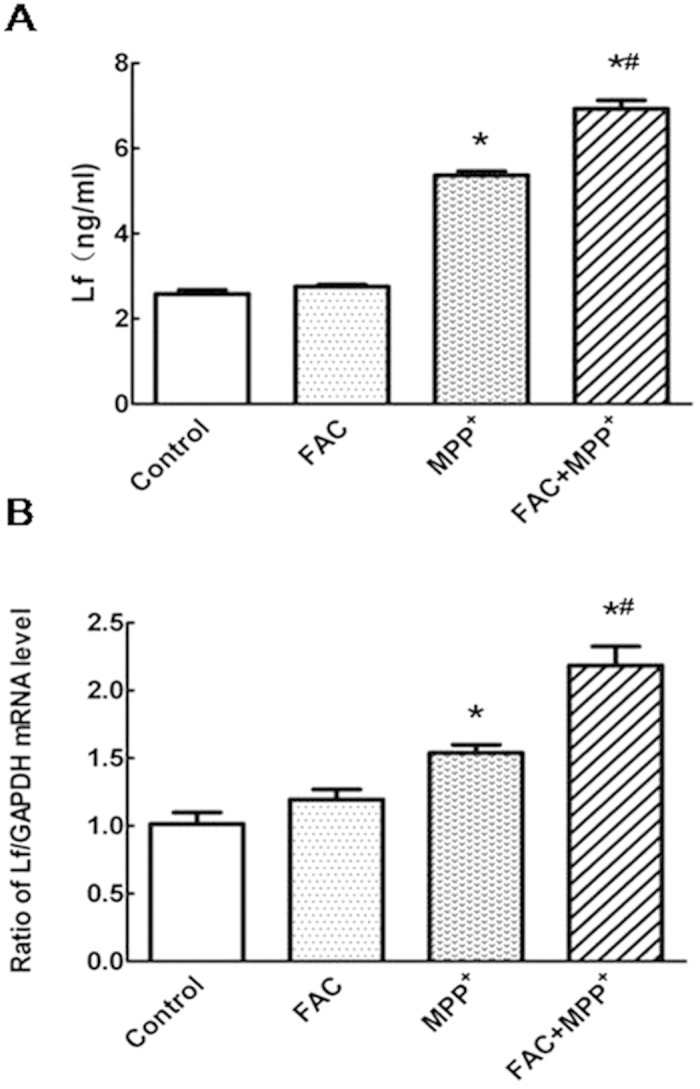
FAC enhanced Lf release and mRNA expression in activated microglia triggered by MPP^**+**^. (**A**) Activation of microglia with MPP^+^ increased Lf release, which was further enhanced by initial FAC incubation. (**B**) Lf mRNA levels in MPP^+^ activated microglia was increased compared with control group. Iron load in microglia elevated the Lf mRNA expression compared with MPP^+^ group. Data are presented as fold changes in Lf mRNA expression with treatment vs control in microglia. *P < 0.05, compared with the control; ^#^*P* < 0.05, compared with MPP^+^ group. n = 6.

We first investigated if cellular iron content could affect the synthesis and release of Lf in activated microglia. Primary microglia were pretreated with 100 μmol/L FAC for 12 h, and then activated by 100 μmol/L MPP^+^ for another 24 h. The conditioned medium from the control or solely FAC treated microglia had barely detectable levels of Lf. Activated microglia by MPP^+^ showed a dramatic Lf release. In the presence of iron, these secretion induced by MPP^+^ was further enhanced (Fig. 1A). Also, we analyzed Lf mRNA levels with real-time PCR (Fig. 1B).Consistent with the Lf secretion in MPP^+^-activated microglia supernatant, Lf mRNA level was increased in MPP^+^-activated microglia, which was further enhanced by FAC pretreatment. This indicated that FAC alone could not increase the expression of Lf in microglia. Both the release and mRNA expression of Lf were increased in MPP^+^ -activated microglia, which could be further enhanced under the condition of iron load.

### Lf antagonized MPP^+^-induced dissipation of ΔΨm

**Figure 2:**
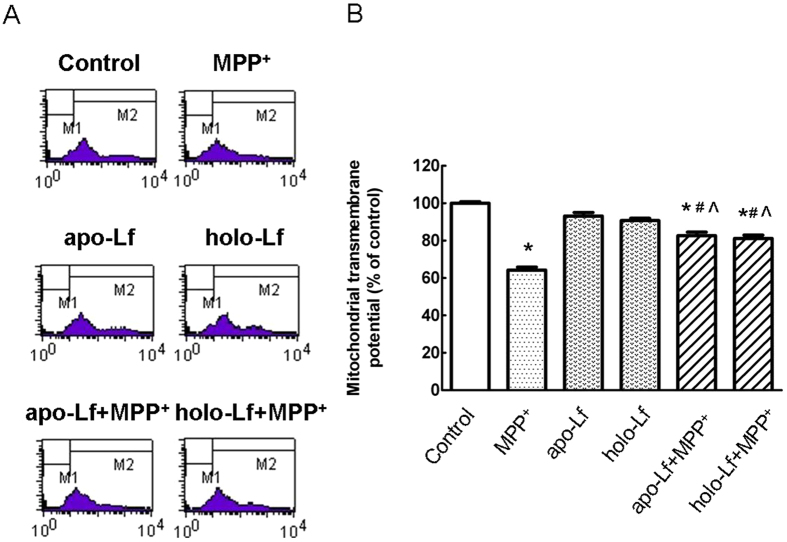
ΔΨm assessed by flow cytometry in MPP^+^-treated VM neurons with apo-Lf or holo-Lf pretreatment. Changes in the mitochondrial membrane potential were measured by rhodamine123 using flow cytometry. (**A**) Representatives of the fluorometric assay on ΔΨm of different groups. Results are shown as FL1-H (fluorescence 1-histogram), setting the gated region M1 and M2 as a marker to observe the changing levels of fluorescence intensity using CellQuest software. Numbers in the M1 gate of the dot-plots show the percentage of apoptotic cells. The ΔΨm was decreased in MPP^+^-treated apoptotic cells and the ratio of M1 area was increased. 100 ng/ml apo-Lf or holo-Lf pretreatment significantly restored the ΔΨm reduction induced by MPP^+^, and the frequency of apoptotic cells was decreased. X mean = mean channel fluorescence of FL-1; Y mean= the number of living cells. (**B**) Statistical analysis. Data were presented as mean ± S.E.M. of three independent experiments. Fluorescence values of the control were set to 100%. *P < 0.05, compared with the control; ^#^*P* < 0.05, compared with apo-Lf group; ^^^*P* < 0.05, compared with holo-Lf group; n = 6.

ΔΨm changes are markers of mitochondria function. We measured the changes of ΔΨm in MPP^+^-treated cells and Lf-pretreated cells. As shown in Fig. 2, when treated with MPP^+^, VM neurons showed a significant decrease of ΔΨm. Pretreatment with Lf partially reversed the decrease in mitochondrial potential. This suggested that Lf could protect cells from MPP^+^-induced oxidative stress by restoring the mitochondria function.

### Lf up-regulated LfR expression in VM neurons

**Figure 3:**
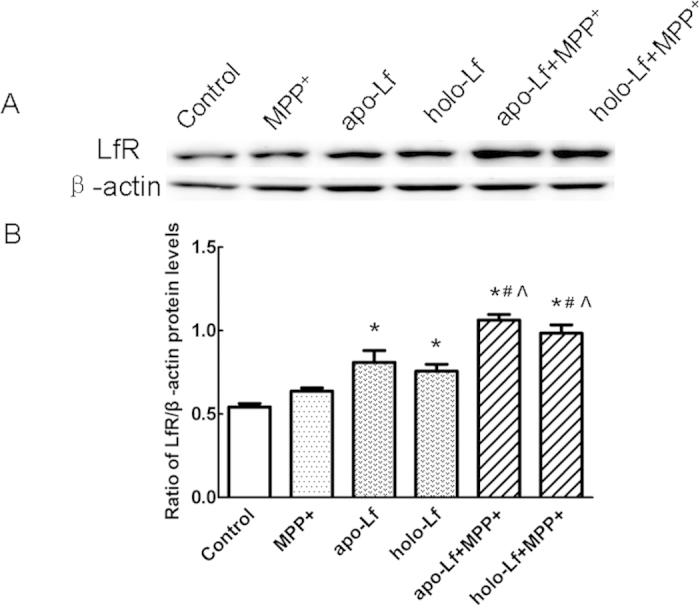
LfR protein levels in MPP^+^-treated VM neurons with apo-Lf or holo-Lf pretreatment. (**A**) Western blots were applied to detect LfR protein levels. LfR expression was increased in apo-Lf or holo-Lf treated VM neurons compared with control group. Apo-Lf or holo-Lf pretreatment increased the expression of LfR in MPP^+^-treated cells. β-actin was used as a loading control. (**B**) Statistical analysis. Data were presented as the ratio of LfR to β-actin. Each bar represented the mean ± S.E.M. of 6 independent experiments. *P < 0.05, compared with the control. ^#^*P* < 0.05, compared with apo-Lf group; ^^^*P* < 0.05, compared with holo-Lf group.

To investigate whether LfR is responsive to Lf treatment, we investigated the expression of LfR in primary cultured VM neurons by Western blots. As shown in Fig. 3, VM neurons endogenously express LfR and there was a significant increase in LfR protein level resulting from the apo-Lf or holo-Lf treatment. This indicated that LfR was abundantly expressed in VM neurons and responsible for the uptake of both apo-Lf and holo-Lf through receptor-mediated endocytosis.

### Blocking LfR could attenuate the protective effects of Lf in MPP^+^-treated VM neurons

**Figure 4:**
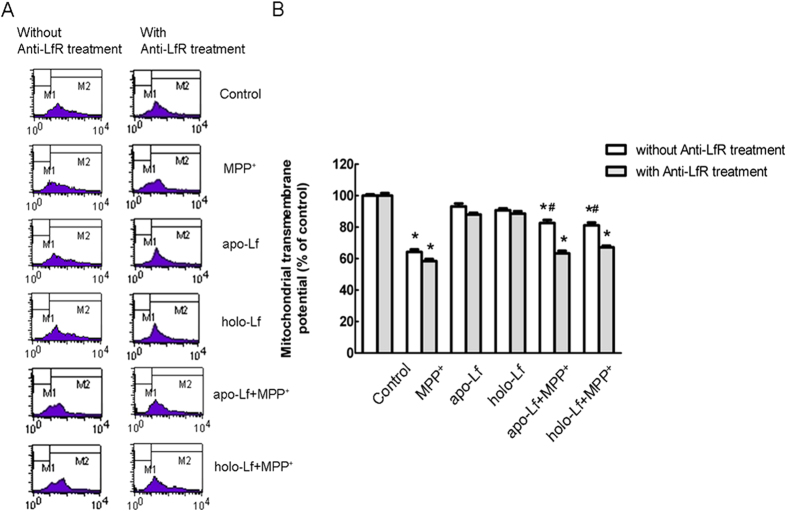
ΔΨm assessed by flow cytometry in VM neurons with blocking LfR. To block binding of ligands to LfR, VM neurons were treated with Anti-LfR for 1 h at 37 °C. Then, VM neurons were treated with 100 ng/ml apo-Lf or 100 ng/ml holo-Lf for 4 h followed by 100 μmol/L MPP^+^ for 24 h. (**A**) Representatives of the fluorometric assay on ΔΨm of different groups. Results are shown as FL1-H (fluorescence 1-histogram), setting the gated region M1 and M2 as a marker to observe the changing levels of fluorescence intensity using CellQuest software. Numbers in the M1 gate of the dot-plots show the percentage of apoptotic cells. The ΔΨm was decreased in MPP^+^-treated apoptotic cells and the ratio of M1 area was increased. 100 ng/ml apo-Lf or holo-Lf pretreatment significantly restored the ΔΨm reduction induced by MPP^+^. Blocking LfR significantly attenuated the protective effect of Lf in VM neurons induced by MPP^+^. X mean = mean channel fluorescence of FL-1; Y mean= the number of living cells. (**B**) Statistical analysis. Data were presented as mean ± S.E.M. of three independent experiments. Fluorescence values of the control were set to 100%. *P < 0.05, compared with the control; ^#^*P* < 0.05, compared with MPP^+^ group; n = 6.

LfR in mediating Lf endocytosis was inhibited by LfR blocking antibody. The results showed that blocking LfR antagonized Lf-induced ΔΨm increase in VM neurons and attenuated the protective effect of Lf on MPP^+^-induced oxidative stress damage (Fig. 4). This directly documented that uptake of both apo-Lf and holo-Lf was inhibited by blocking LfR and LfR played a major role in apo-Lf and holo-Lf endocytosis into VM neurons.

### Lf induced up-regulation of Cu/Zn-SOD in VM neurons

**Figure 5:**
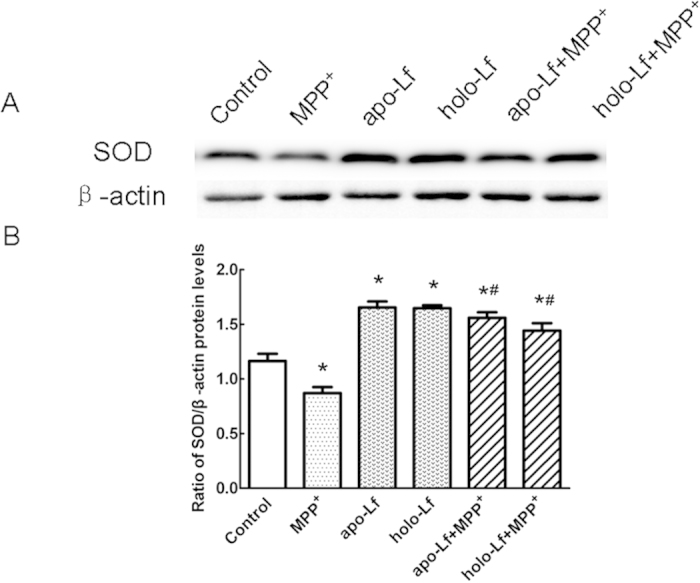
Cu/Zn–SOD protein levels in MPP^+^-treated VM neurons with apo-Lf or holo-Lf pretreatment. (**A**) Western blots were applied to detect Cu/Zn–SOD protein levels. Cu/Zn–SOD protein level was down-regulated in VM neurons by MPP^+^. Apo-Lf or holo-Lf increased the expression of Cu/Zn–SOD in VM neurons compared with the control group. Apo-Lf or holo-Lf pretreatment reversed the decrease of Cu/Zn–SOD expression in MPP^+^-treated cells. β-actin was used as a loading control. (**B**) Statistical analysis. Data were presented as the ratio of Cu/Zn–SOD to β-actin. Each bar represented the mean ± S.E.M. *P < 0.05, compared with the control; ^#^*P* < 0.05, compared with MPP^+^ group; n = 6.

We examined Cu/Zn-SOD expression, a highly potent protective agent against cell injury during oxidative stress in VM neurons. In 100 μmol/L MPP^+^-treated cells, Cu/Zn-SOD protein level was down-regulated by 32% of control. However, after 100 ng/ml apo-Lf or 100 ng/ml holo-Lf pretreatment for 4 h, the protein levels could be up-regulated significantly (Fig. 5). This indicated that both apo-Lf and holo-Lf could exert neuroprotective effects against MPP^+^-induced damage by a mechanism, believed to increase the level of Cu/Zn-SOD and anti-oxidative stress.

### Lf induced up-regulation of Bcl-2/Bax ratio in VM neurons

**Figure 6:**
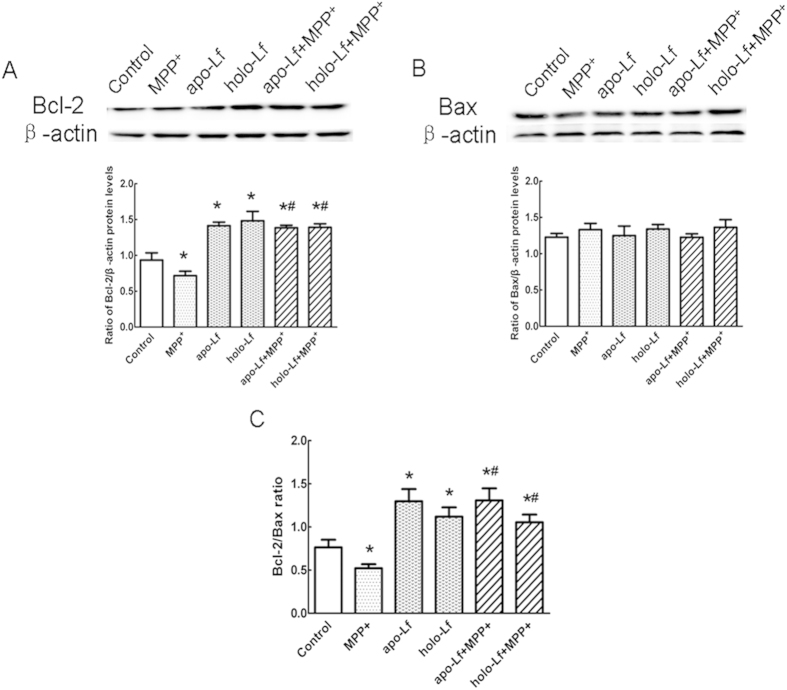
Bcl-2 and Bax protein levels in MPP^+^-treated VM neurons with apo-Lf or holo-Lf pretreatment. Western blots were applied to detect Bcl-2 and Bax protein levels. (**A**) Bcl-2 expression was decreased in MPP^+^-treated cells. Both apo-Lf and holo-Lf could elevate Bcl-2 expressions in VM neurons compared with control group. Apo-Lf or holo-Lf pretreatment increased the expression of Bcl-2 in MPP^+^-treated cells. (**B**) Bax protein levels had no change in different groups. (**C**) Effects of Lf on Bcl-2/Bax ratio in different groups. Bcl-2/Bax ratio significantly increased due to apo-Lf or holo-Lf treatment in VM neurons. And apo-Lf or holo-Lf pretreatment reversed the decrease of Bcl-2/Bax ratio in MPP^+^ treated group. β-actin was used as a loading control. Data were presented as the ratio of Bcl-2 or Bax to β-actin. Each bar represented the mean ± S.E.M. *P < 0.05, compared with the control; ^#^*P* < 0.05, compared with MPP^+^ group; n = 4.

Apoptotic pathway plays an important role in neuronal injury^17^. Extensive evidence suggests that the Bcl-2 family of proteins forms a complex network to regulate apoptosis. The anti-apoptotic protein Bcl-2 and the pro-apoptotic protein Bax can migrate from the cytoplasm to mitochondria, which are distributed in a manner that is consistent with mitochondrial release of cytochrome C and caspase^18^. Treatment of VM neurons with 100 μmol/L MPP^+^ for 24 h resulted in a decreased protein level of Bcl-2 ,and this effect could be significantly reversed by 100 ng/ml apo-Lf or 100 ng/ml holo-Lf pretreatment for 4 h (Fig. 6A). Nevertheless, there were no obvious changes in Bax protein levels (Fig. 6B). This indicated that both apo-Lf and holo-Lf inhibited cellular apoptosis after MPP^+^-induced injury by upregulating Bcl-2/Bax ratio (Fig. 6C), suggesting that the mitochondrial apoptotic pathway is involved in the protective effects of Lf against MPP^+^-induced neuronal injury.

### Apo-Lf but not holo-Lf accounted for iron chelation in VM neurons

**Figure 7:**
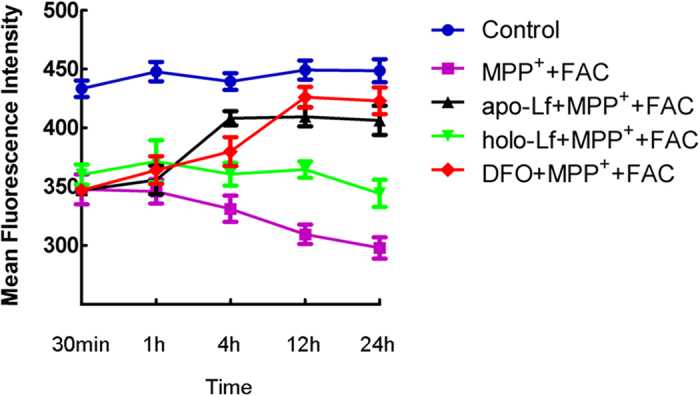
Apo-Lf but not holo-Lf reduced the cellular LIP. LIP in VM neurons was determined by the fluorescence intensity of calcein, an indicator of intracellular pool of free iron. The fluorescence intensity represented the mean value of VM neurons at each time point and was presented as the mean ± S.E.M. of six independent experiments. Results were carried out by two-way ANOVA followed by Student-Newman-Keuls test. There was a significant decrease in the fluorescence intensity in neurons treated with MPP^+^+ FAC compared with the control, indicating increased free iron level. Fluorescence intensities increased in apo-Lf+ MPP^+^+FAC or DFO+MPP^+^+FAC groups, indicating decreased free iron levels in these cells. There was no obvious reverse in fluorescent intensity in holo-Lf+ MPP^+^+FAC group, indicating holo-Lf did not change the cellular iron levels (two-way ANOVA, F = 39.835, *P* < 0.05, apo-Lf+MPP^+^+FAC vs MPP^+^+FAC; *P* < 0.05, DFO+MPP^+^+FAC vs MPP^+^+FAC; *P* < 0.05, holo-Lf+MPP^+^+FAC vs apo-Lf+MPP^+^+FAC and DFO+MPP^+^+FAC;*P* > 0.05, holo-Lf+MPP^+^+FAC vs MPP^+^+FAC).

LIP is localized primarily, but not exclusively, in the cytosol and is regarded as the crossroad of cellular iron traffic. In this study, DFO was selected as iron chelator because of its higher affinity for the ferric ion and its well-documented use in iron chelation studies^14^. To elucidate whether apo-Lf or holo-Lf could affect intracellular iron transport, we used fluorescence dye calcein to measure cellular LIP in VM neurons. As shown in Fig. 7, there was a steady intracellular fluorescence quenching in control group. In 100 μmol/L MPP^+^ and 100 μmol/L FAC treated group, there was a more dramatic decrease in fluorescent intensity compared with the control. This indicated that MPP^+^ caused more iron accumulated in VM neurons. When cells were incubated with 100 ng/ml apo-Lf or 100 μmol/L DFO for 30 min, 1 h, 4 h, 12 h, 24 h respectively, we observed apo-Lf could significantly reverse the fluorescence intensity from 4 h, which was much earlier than that of DFO. The higher fluorescence intensity confirmed decreased iron levels in apo-Lf or DFO treated VM neurons. However, there were no obvious changes in fluorescent intensity with 100 ng/ml holo-Lf treatment. This indicated that LIP had no change after holo-Lf treatment. All the results from calcein indicated that MPP^+^ could enhance iron level in primary cultured VM neurons. Apo-Lf but not holo-Lf was therefore highly efficient at binding iron, reducing neuronal iron accumulation. Thus, apo-Lf but not holo-Lf had iron chelating activity.

## Discussion

The present study first showed that cellular iron homeostasis influenced the synthesis of Lf in activated microglia. Lf could significantly antagonize MPP^+^-induced neurotoxicity in primary cultured VM neurons of rats, rather than to transport excessive iron to neurons leading to cell damages. The protective effect of Lf does not appear to be connected with its iron binding ability.

McGeer and coworkers first described increased number of microglia in the SN of post-mortem PD patients^19^. Microglia, often referred to as the resident immune cells, play a key role in the process of PD. SN contains a higher proportion of microglia than other brain regions^20^. Microglia are readily activated by an extensive list of stimuli, such as LPS, MPP^+^, pesticides (e.g., paraquat and rotenone), disease proteins (e.g. Aβ and α-synuclein)^21^. Activated microglia releases pro-inflammatory factors that may be toxic to neurons. However, it can also release neurotrophins and may have neurotrophic effects^22^. Double immunofluorescent labeling confirmed that the two Lf immunostained cell populations were activated microglia and dopaminergic neurons. Since activated microglia contained both Lf and its messenger, these cells are the Lf producing cells. While no Lf messengers were found in dopaminergic neurons, indicating that Lf released by activated microglia enter neurons through LfR^4^. Our results showed that FAC alone could not increase the expression of Lf in microglia. Both the release and mRNA expression of Lf were enhanced in activated microglia induced by MPP^+^. The results were similar with Zhang’s data that iron loading alone does not increase the release of the cytokine. Activation with LPS increases the cytokine release and the amounts of cytokines were enhanced if the microglial were initially over-loaded by iron^23^. But the reason that iron status of the microglia influence the Lf production has not been determined, but the data are compelling that microglia function is iron dependent. On the basis of our data, we propose that nigral iron accumulation not only influence dopaminergic neuron survival directly, but affects microglia function thus influence nigral neurons indirectly. Several lines of evidence indicate that alterations in iron metabolism, excessive oxidative stress, and/or immune-mediated pathophysiology might contribute to neuronal loss of PD. Lf acts both as an iron-binding protein and a protective factor in the inflammation process, oxidative reactions and etc. It is necessary to elucidate whether Lf acts as an iron donor, leading to oxidative damage in vulnerable neurons or represent a protective factor. Lf synthesized as an iron free molecule (apo-Lf) in activated microglia^4^. In this study, we observed that apo-Lf was protective to VM neurons against MPP^+^ toxicity. Even when Lf was iron saturated as a holo-Lf, the protective function of Lf still existed. The most striking characteristic of Lf is its high affinity for iron. The affinity of Lf for iron is 300 times higher than that of transferrin^24^, because Lf binds iron even at pH 3.5 while transferrin releases iron no lesser than PH 5. Therefore, apo-Lf may act as a powerful iron chelator, just as the results in this study have showed: apo-Lf, as well as DFO could chelate iron in VM neurons, resulting in a decrease in LIP, which is a pool of chelatable and redox-active iron. Holo-Lf is iron saturated that could not chelate iron anymore, but holo-Lf is not toxic to the VM neurons, because it binds iron very tightly that no iron was released to the LIP. Lf might function as a major specialized iron scavenger and acts as an antioxidant rather than an iron donor. Sequestration of free iron by Lf may inhibit the iron-catalyzed formation of hydroxyl radicals, and the presence of Lf at sites where oxidative stress occurs may limit cell damage. There were no difference between apo-Lf and holo-Lf on the neuro-protective function, indicating that the Lf protects the VM neurons against MPP^+^ does not appear to be connected with its iron binding ability.

LfR expression is responsive for the Lf level. In our experiment, LfR was increased after Lf treatment. Therefore, high levels of Lf in the surviving nigral neurons may be the result of an increase in the expression of LfR, which bind with extracellular Lf, possibly from microglia or from the blood. In contrast to transferrin, the binding of Lf to the LfR is independent of its degree of iron saturation^25^. The capture of extracellular Lf may be responsible for an excessive accumulation of iron in susceptible cells, although the iron binding with Lf is not toxic. In addition, in degenerating nigral neurons, iron has been shown to accumulate principally in vesicular compartments^26^, which is consistent with the Lf-staining patterns^4^. LfR internalizes Lf, and LfR is expressed at the cell surface of platelets, megacaryocytes, enterocytes, dopaminergic neurons, and mesencephalon microvessels. LfR is a major pathway through which Lf is taken up by enterocytes, which occurs independently of iron saturation through clathrin-mediated endocytosis^27^. In addition, lipoprotein receptor-related protein (LRP) displays a high affinity for Lf^28^,^29^. LRP might be involved in this transcellular transport across the brain endothelium. LRP has also been studied in the central nervous system and was found associated with neurons, weakly on some glial cells, and discontinously along the membranes of the capillaries^30^. LRP might therefore be responsible for Lf accumulation in some specific brain areas. Drug delivery to the brain is limited by the presence of the blood-brain barrier (BBB). Lf, with its receptor expressed on brain BBB, is transported across the BBB. Therefore, Lf is used as a brain targeting molecule resulted in successful brain delivery^31^. Lf was conjugated to PEG-PLGA nanoparticles (NP) to construct a novel brain drug delivery system (Lf-NP). This Lf-NP was tested to be effective for the treatment of PD animals^32^ and glioma^33^, demonstrating that Lf-NP was a promising brain drug delivery system with reasonable toxicity.

MPTP is a well established pro-toxin that causes the selective destruction of the SN in humans and other primates resulting in an acute Parkinsonism^34^,^35^. MPTP is converted to its active MPP^+^ form by the monoamine oxidase B and then taken up by dopaminergic neurons through the terminal receptor dopamine transporter. Upon entering the dopaminergic neurons, MPP^+^ will selectively damage mitochondria function and lead to cell death. Oxidative stress is defined as an imbalance between the production of free radicals and antioxidant defence^36^. Oxidative stress plays a key role in PD^37^, and dopamine-rich areas of the brain are particularly vulnerable to oxidative stress. The Bcl-2 family of proteins participates in intracellular apoptotic signal transduction by regulating the permeability of the mitochondrial membrane. Bcl-2 can inhibit apoptosis by binding to the pro-apoptotic Bax, it is believed that the Bcl-2/Bax ratio is a determining factor for the cell’s fate^38^. Both apo-Lf and holo-Lf treated VM neurons showed an increased level of ΔΨm, Cu/Zn-SOD and Bcl-2/Bax ratio, rescuing neurons from MPP^+^-induced toxicity. Some reports show that Lf protected PC12 cells from FasL-induced apoptosis through extracellular-signal-regulated kinase 1/2 (ERK1/2) phosphorylation pathway^39^. Lf increased calcium shuttling between ER and mitochondria via a PI3K-AKTdependent mechanism^7^. Further experiment showing the downstream pathways for Lf neuroprotection against MPP^+^ need to be established.

In conclusion, we demonstrate that the expression of Lf is up-regulated in activated microglia under the condition of iron load. Lf can antagonize MPP^+^-induced neurotoxicity to VM neurons by mechanisms, believed to enhance the ΔΨm, improve the activity of Cu/Zn-SOD and increase the expression of Bcl-2. The protective effect of Lf on VM neurons against MPP^+^ does not appear to be connected with its iron binding ability. Finally, the present study opens the eventuality to explore the possible use of Lf as a therapeutic approach in PD.

## Additional Information

**How to cite this article**: Wang, J. *et al.* The protective effect of lactoferrin on ventral mesencephalon neurons against MPP^+^ is not connected with its iron binding ability. *Sci. Rep.* **5**, 10729; doi: 10.1038/srep10729 (2015).

## Supplementary Material

Supplementary Information
