# Supplementary material for: The protective effect of lactoferrin on ventral mesencephalon neurons against MPP+ is not connected with its iron binding ability
Source: Sci Rep. 2015 Jun 2;5:10729. doi: 10.1038/srep10729 (PMC4451802; doi:10.1038/srep10729)
Supplement: Supplementary Information [file srep10729-s1.pdf]

The protective effect of lactoferrin on ventral mesencephalon neurons against MPP^+ is not connected with its iron binding ability

Jun Wang*[1], Mingxia Bi[1], Huiying Liu, Ning Song, Junxia Xie**

Department of Physiology, Shandong Provincial Key Laboratory of Pathogenesis and Prevention of Neurological Disorders, Shandong Provincial Collaborative Innovation Center for Neurodegenerative Disorders and State Key Disciplines: Physiology, Medical College of Qingdao University, Qingdao 266071, China

[1]Co-first author

* Corresponding author. Tel.: +86 532 83780051; fax: +86 532 83780136.

**Co-corresponding author. Tel.: +86 532 85955891; fax: +86 532 85953085.

E-mail addresses: junwangqdu@163.com (J. Wang)

jxiaxie@public.qd.sd.cn (J. Xie)

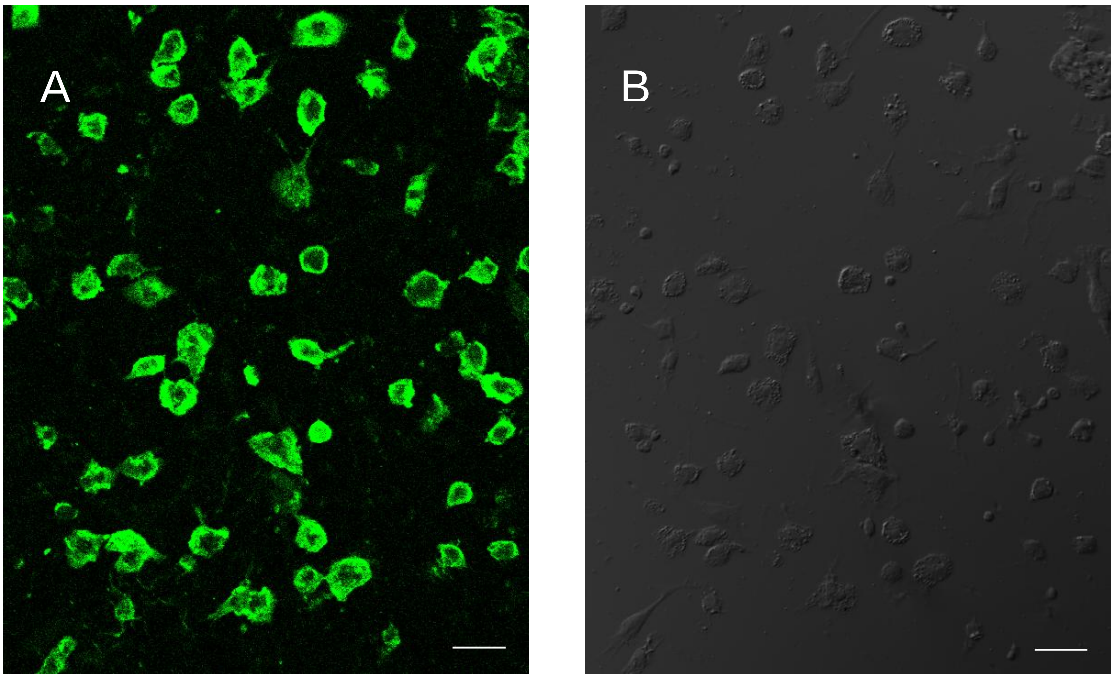

Supplementary Figure S1. Immunofluorescence labeling of primary microglia with CD11b

CD11b is the marker used to identify primary cultured microglia. Images of immunofluorescence labeling (A) and phase contrast (B) showed at least 95% CD11b positive cells in the microglia culture. Scale bar=25**μ**m.

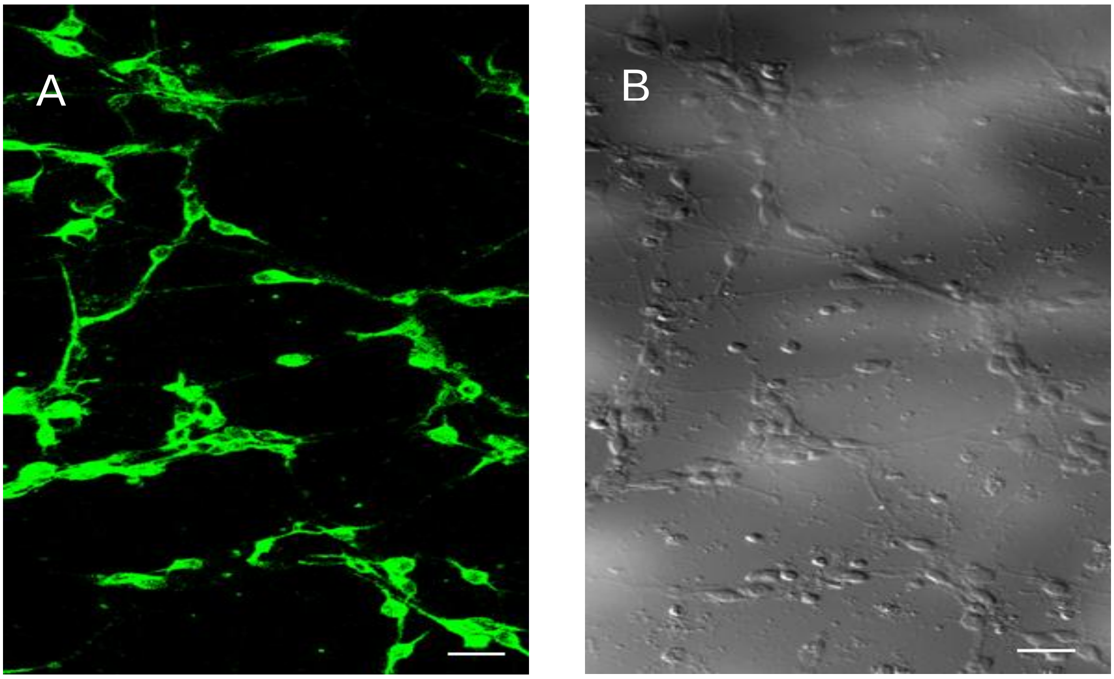

Supplementary Figure S2. Immunofluorescence labeling of primary VM neurons with MAP2

MAP2 is the marker used to identify primary cultured microglia. Images of immunofluorescence labeling (A) and phase contrast (B) showed at least 95% MAP2 positive cells in the VM neurons culture. Scale bar=25μm .
